# Simulation of Organic Liquid Product Deoxygenation through Multistage Countercurrent Absorber/Stripping Using CO_2_ as Solvent with Aspen-HYSYS: Process Modeling and Simulation

**DOI:** 10.3390/molecules27072211

**Published:** 2022-03-29

**Authors:** Manoel Raimundo dos Santos Junior, Elinéia Castro Costa, Caio Campos Ferreira, Lucas Pinto Bernar, Marcilene Paiva da Silva, Andréia de Andrade Mâncio, Marcelo Costa Santos, Sílvio Alex Pereira da Mota, Douglas Alberto Rocha de Castro, Sergio Duvoisin Junior, Luiz Eduardo Pizarro Borges, Marilena Emmi Araújo, Nélio Teixeira Machado

**Affiliations:** 1 Graduate Program of Natural Resources Engineering of Amazon, Campus Profissional-UFPA, Rua Augusto Corrêa N° 1, Belém 66075-110, Brazil; manoeljr@ufpa.br (M.R.d.S.J.); elineia.costa.ec@gmail.com (E.C.C.); caiocf7@hotmail.com (C.C.F.); lucas.bernar7@gmail.com (L.P.B.); dedeiaam@yahoo.com.br (A.d.A.M.); silviomota@unifespa.edu.br (S.A.P.d.M.); douglascastro87@hotmail.com (D.A.R.d.C.); 2 Graduate Program of Chemical Engineering, Campus Profissional-UFPA, Rua Augusto Corrêa N° 1, Belém 66075-900, Brazil; arci_paiva@hotmail.com (M.P.d.S.); marcelo.santos@ufra.edu.br (M.C.S.); meaaraujo@gmail.com (M.E.A.); 3 Faculty of Chemical Engineering, Universidade do Estado do Amazonas-UEA, Avenida Darcy Vargas N° 1200, Manaus 69050-020, Brazil; sjunior@uea.edu.br; 4 Laboratory of Catalyst Preparation and Catalytic Cracking, Section of Chemical Engineering, Instituto Militar de Engenharia-IME, Praça General Tibúrcio N° 80, Rio de Janeiro 22290-270, Brazil; luiz@ime.eb.br; 5 Faculty of Sanitary and Environmental Engineering, Campus Profissional-UFPA, Rua Augusto Corrêa N° 1, Belém 66075-110, Brazil

**Keywords:** OLP, deoxygenation, absorber columns, process flowsheet, process simulation, Aspen-HYSYS

## Abstract

In this work, the deoxygenation of organic liquid products (OLP) obtained through the thermal catalytic cracking of palm oil at 450 °C, 1.0 atmosphere, with 10% (wt.) Na_2_CO_3_ as a catalyst, in multistage countercurrent absorber columns using supercritical carbon dioxide (SC-CO_2_) as a solvent, with an Aspen-HYSYS process simulator, was systematically investigated. In a previous study, the thermodynamic data basis and EOS modeling necessary to simulate the deoxygenation of OLP was presented. This work addresses a new flowsheet, consisting of 03 absorber columns, 10 expansions valves, 10 flash drums, 08 heat exchanges, 01 pressure pump, and 02 make-ups of CO_2_, aiming to improve the deacidification of OLP. The simulation was performed at 333 K, 140 bar, and (S/F) = 17; 350 K, 140 bar, and (S/F) = 38; 333 K, 140 bar, and (S/F) = 25. The simulation shows that 81.49% of OLP could be recovered and that the concentrations of hydrocarbons in the extracts of absorber-01 and absorber-02 were 96.95 and 92.78% (wt.) on a solvent-free basis, while the bottom stream of absorber-03 was enriched in oxygenated compounds with concentrations of up to 32.66% (wt.) on a solvent-free basis, showing that the organic liquid products (OLP) were deacidified and SC-CO_2_ was able to deacidify the OLP and obtain fractions with lower olefin contents. The best deacidifying condition was obtained at 333 K, 140 bar, and (S/F) = 17.

## 1. Introduction

Thermal catalytic cracking is one of the most promising processes with which to convert and/or transform vegetable oils [1,2,3,4,5,6,7,8,9], residual oils [10,11], animal fats [12,13,14], mixtures of carboxylic acids [14,15,16], soaps of carboxylic acids [17], scum, grease, fats [18,19,20], and residual animal fat [13] into liquid hydrocarbon-based biofuels [7,8,9,17,18,19,20]. This process has the objective to obtain liquid hydrocarbons for use as fuels [1,2,5,7,8,9,10,12,13,14,15,16,17,18,19,20].

The reaction products obtained by the thermochemical transformation of vegetable oils, residual oils, animal fats, mixture of carboxylic acids, scum, grease, and fats, soaps of carboxylic acids, and residual animal fats include gaseous and liquid fuels, water, aqueous acid phases, and coke [3,4,5,7,8,9,14,15,16,17,18,19,20]. The physicochemical properties and chemical composition of organic liquid products depends on the compositional and physicochemical properties of the raw materials, process temperatures, residence time, mode of operation (fluidized bed reactor, sludge bed reactor, etc.), presence of water in the raw material, and catalyst nature (acid, basic) selectivity [1,3,4,5,9,10,11,14,18].

The organic liquid products are composed of alkanes, alkenes, ring-containing alkanes, ring-containing alkenes, cyclo-alkanes, cyclo-alkenes, and aromatics [1,5,7,8,9,12,14,15,17,18,19,20], as well as oxygenates, including carboxylic acids, aldehydes, ketones, fatty alcohols, and esters [1,3,4,5,7,8,9,12,14,17,18,19,20].

The organic liquid products obtained through the catalytic cracking of lipid base raw materials, including palm oil [7,8,9], waste oils [10,11], carboxylic acids [14], animal fats [14], soaps of carboxylic acids [17], as well as the synthetic carboxylic acids derivates of methyl octanoate [21], using sodium salts as basic catalysts (e.g., Na_2_CO_3_), as well as catalysts with basic properties, such as X zeolites (e.g., KNaX1, CsNaX1) produced from FAU zeolites, SBA-15 ordered mesoporous silicate, and clays (sepiolite and hydrotalcite) impregnated with cesium [21], present low concentrations of carboxylic acids [7,8,9,14,17,21] due to the catalyst’s activity in the secondary cracking step, whereas the carboxylic acids are broken up to produce hydrocarbons [22]. The organic liquid products can be refined and/or upgraded by applying physical processes (filtration, decantation, and centrifugation) [7,8,9,17,18,19,20] and thermal separation processes, including liquid–liquid extraction [23,24], distillation [7,8,9,14,17,18,19,20,24,25,26], and adsorption [24], to produce high-quality green fuel-like fractions with the potential to partially substitute fossil fuels. The disadvantages of organic liquid products obtained through the catalytic cracking of lipid base raw materials, including vegetable oils, residual oils, animal fats, mixture of carboxylic acids, scum, grease, and fats, soaps of carboxylic acids, and residual animal fats, are the remainder of the high acid values, especially if catalysts with acid properties and/or characteristics are used [3,4,16,18,19,20], and the high concentrations of olefins, making fuels thus derived corrosive and unstable [7].

In recent years, thermal separation processes have been applied to remove and/or recover the oxygenated compounds from organic liquid products, particularly through fractionation by using single-stage and multistage distillation to obtain hydrocarbon-like fuels in the temperature boiling point range of gasoline, kerosene, and diesel-like fractions [3,4,7,8,9,12,14,17,18,19,20,24,25,26], as well as oxygenates from biomass-derived bio-oils by applying separation and purification processes, including molecular distillation [27,28,29], fractional distillation [30,31,32,33], and liquid–liquid extraction [23,24,34]. However, until now, no systematic study has been reported in the literature related to the simulation of organic liquid product (OLP) fractionation and/or purification in multistage countercurrent absorber/stripping columns using supercritical CO_2_ as a solvent and using the chemical process simulator Aspen-HYSYS.

Multistage gas extraction in countercurrent columns has been considered to be an alternative separation technique for the extraction and fractionation of liquid mixtures with potential applications in the food industry [35,36,37]. The applications include the removal of terpenes from citrus oils [38,39], the isolation of polycyclic aromatic hydrocarbon (PAH) oligomers [40], the deacidification of olive and rice bran oils [41,42], the fractionation of fatty acids from palm fatty acid distillates [43,44], the purification of fat-soluble substances (tocopherols, sterols, carotenes, and squalene) from palm oil, olive oil, soya deodorize distillates, olive oil deodorize distillates, and hexane extracts of olive leaves [41,45], the separation of aroma constituents from aqueous solutions of apple, brandy, and wine-must aroma [46], and the fractionation of the model mixture of squalene/methyl oleate [47,48], among other applications [49,50,51,52,53,54,55,56,57].

Despite the wide range of applications of multistage gas extraction in countercurrent packed columns, particularly in the food industry, as reported in the reviews by Brunner and Bejarano et al. [36,49], only a small number of works are devoted to the simulation of multistage gas extraction using self-made computer codes or commercial chemical process simulators, such as Aspen-HYSYS, as described chronologically in what follows [50,51,52,53,54,55,56,57,58,59].

Moricet [50], in a pioneering computer simulation study in the early 1980s, developed a self-made computer code in Fortran to simulate the separation of mono-glycerides from a model mixture of oleic acid/glycerides, as well as the separation of fatty acids from palm oil in countercurrent columns using carbon dioxide as solvent.

Simões and Brunner [51] applied high-pressure phase equilibrium data for a system of olive oil/CO_2_ to simulate a countercurrent packed column for the deacidification of olive oil by using a staged equilibrium model self-made program, and the simulations were compared with experimental mass transfer data.

Mendes et al. [52] studied the concentration of tocopherols from soybean oil deodorizer distillate, represented as a synthetic mixture of tocopherol/squalene/carboxylic acids, by simulating the separation of tocopherol from a synthetic mixture of tocopherol/squalene/carboxylic acids using SC-CO_2_ as a solvent within a flowsheet consisting of a one-stage extractor and a flash separator using the commercial simulator ASPEN+. The PR-EOS with the LCVM mixing rule thermodynamic fluid package was used to corroborate the equilibrium data of the system soybean oil deodorizer distillate/CO_2_ [53].

Benvenuti et al. [54] used experimental data for a semi-continuous single-stage extractor to remove the terpenes from lemon essential oil using SC-CO_2_ as a solvent to model the process by assuming an equilibrium between the coexisting gaseous phase at the exit of single-stage apparatus and the liquid phase inside it, being the equilibrium described by the PR-EOS, using a self-made academic computer. The thermodynamic modeling of the system extended to study the steady-state simulation of a multistage column with the recycling of the solvent was represented by a flowsheet consisting of a series of flash separators using the PR-EOS with quadratic mixing rules as the thermodynamic fluid package.

Moraes et al. [55] simulated the recovery of provitamin A from esterified palm oil using a mixture of SC-CO_2_/ethanol as a solvent with the commercial simulator HYSYSTM, adapting the process units to the typical operating conditions of SFE. Because esterified palm oil is a complex mixture, it was necessary to insert the compounds as hypotheticals to predict/estimate the physical and thermo-physical properties using the UNIFAC group contribution. The optimization performed for each unit of the process was based on state conditions (T, P) to obtain maximum carotenes recovery. The carotenes concentrated with high yields using 02 SFE cycles, and the ethyl esters were present as by products.

Vásquez et al. [42] applied GC-EOS to simulate the separation process and for the design of experimental conditions. The thermodynamic model was used to obtain optimal process conditions and to enhance squalene recovery, including the reflux of extracts and the recirculation of SC-CO_2_ in a continuous countercurrent extraction column.

Fernandes et al. [47] developed a dynamic model of the SFE process applied to the fractionation of a binary mixture of squalene/methyl oleate using SC-CO_2_. The flowsheet, modularly organized into a set of sub-models, includes a countercurrent packed column, a separator, a heat exchanger, and the same make-up gas of CO_2_, showing good agreement between experimental and predicted results. The model correctly predicts the outlet stream composition profiles of all the case studies.

Fernandes et al. [48] developed a non-isothermal dynamic model to simulate the fractionation of a binary mixture of squalene/methyl oleate with SC-CO_2_ in a countercurrent column with structured packing. The model solves the momentum and energy balances within the packed column. The coexisting liquid–gaseous phases were assumed to be in local thermal equilibrium, showing good agreement between the measured and predicted temperature and the composition profiles of the gas and liquid phases, along with the column. The model was also applied to determine the optimal extraction conditions that maximize squalene recovery.

Fornari et al. [41] applied GC-EOS to simulate the deacidification of olive oil and the recovery of minor lipid compounds in a countercurrent packed column using SC-CO_2_. The GC-EOS model was used to represent phase equilibria of the multicomponent system oil/CO_2_, as well as to simulate and optimize the SFE process.

Vásquez et al. [42] applied GC-EOS to simulate the deacidification of olive oil in a countercurrent column using SC-CO_2_. The olive oil was represented by a binary model mixture of oleic acid/triolein, showing a satisfactory agreement between the experimental and computed yields, as well as the carboxylic acid content in the raffinates.

Da Silva [58] simulated the fractionation of liquid mixtures, including the deacidification of olive oil and the enrichment of fat-soluble substances from soya deodorize distillates, in a countercurrent column using SC-CO_2_ with the chemical process simulator Aspen-HYSYS.

Fernandes et al. [56] developed a complete non-isothermal dynamic model to simulate an SFE plant. The flowsheet was modularly organized into a set of sub-models of the main unit operations, including the countercurrent packed column operating with reflux, a compressor, a heat exchanger, a separator, and the CO_2_ make-up. The modules were interconnected by appropriate boundary conditions that couple the mass, momentum, and energy equations. The model showed good agreement between the experimental and predicted results.

Pieck et al. [57] simulated extract and raffinate compositions and gas loadings through the fractionation of water/ethanol mixtures in countercurrent columns using SC-CO_2_ as a solvent, at the laboratory, pilot, and industrial scales, by applying thermodynamic, mass transfer, and hydrodynamics models, as well as an equilibrium-stage model. The main objective was to contribute a sizing methodology for a countercurrent column using SC-CO_2_ as a solvent.

Costa et al. [59] simulated the SC-CO_2_ fractionation of organic liquid products (OLP), obtained through the catalytic cracking of palm oil at 450 °C, 1.0 atm, using 10% (wt.) Na_2_CO_3_ as a catalyst. The simulations were performed by selecting the multistage countercurrent absorber/stripping unit operation column using SC-CO_2_ as a solvent with the help of the chemical process simulator Aspen-HYSYS 8.6 (Aspen One, 2015) at 333 K and 140 bar and 333 K and 180 bar, with solvent-to-feed ratios (S/F) = 12, 15, 17, 25, 30, and 38, applying flowsheets with one absorber column and two absorber columns. The best deacidifying condition was obtained at 333 K, 140 bar, and (S/F) = 17, presenting a top stream yield of 36.65% with 96.95% (wt.) hydrocarbons and 3.05% (wt.) oxygenates, showing a decrease in the oxygenates content in the OLP feed from 10% (wt.), with 2.63% (wt.) carboxylic acids, to 3.05% (wt.) oxygenates, with 0.52% (wt.) carboxylic acids. 

The objective of this work was to apply the chemical process simulator Aspen-HYSYS 8.6 (Aspen One, 2015) to simulate the SC-CO_2_ deoxygenation/deacidification of the organic liquid products (OLP) obtained through the catalytic cracking of palm oil at 450 °C, 1.0 atm, using 10% (wt.) Na_2_CO_3_ as a catalyst [9]. The simulations were performed by selecting the multistage countercurrent absorber/stripping unit operation column. A new flowsheet was created that consisted of 03 absorber columns, 10 expansion valves, 10 flash drums, 08 heat exchanges, 01 pressure pump, and 02 make-ups of CO_2_, aiming to improve the deacidification of OLP. The simulation was performed at 333 K, 140 bar, and (S/F) = 17; 350 K, 140 bar, and (S/F) = 38; 333 K, 140 bar, and (S/F) = 25. The process performance was evaluated by analyzing the yield and recovery of hydrocarbons, olefins, oxygenates, carboxylic acids in the top and bottom streams, as well as the feasibility of deacidifying OLP fractions with a lower content of alkenes (olefins).

## 2. Methodology

### 2.1. Simulation Methodology

The organic liquid products (OLP) obtained through the thermal catalytic cracking of palm oil at 450 °C, 1.0 atm, using 10% (wt.) Na_2_CO_3_ as a catalyst, at a pilot scale, are a complex mixture of hydrocarbons (alkanes, alkenes, cycloalkanes, and aromatics) and oxygenates (carboxylic acids, ketones, and alcohols) [9]. Since high-pressure phase equilibrium data for the binary pairs of OLP-compounds-i/CO_2_ are scarce, the chemical composition of OLP determined through GC-MS identified approximately 90% (area.) hydrocarbons and 10% (area.) oxygenates, and found that acidity is mainly due to the presence of carboxylic acids, whereby OLP has been described by the key compounds of hydrocarbons (*decane*, *undecane*, *tetradecane*, *pentadecane*, *hexadecane*, and *octadecane*) and oxygenates (*palmitic acid*, *oleic acid*) [59,60]. The simulations were performed by selecting the unit operation multistage countercurrent absorber, as the dissolution/solubilization of gases (CO_2_) in liquids (OLP) is a function of state conditions (P, T), whereby a fraction of the coexisting liquid phase dissolves in the gas phase, such as with a gas (CO_2_)–liquid (OLP) equilibrium behavior. In the multistage countercurrent absorber column, the fraction collected at the top stream is the extract, that is, a phase rich in SC-CO_2_, containing the more soluble compounds, in this case, the hydrocarbons (*decane*, *undecane*, *tetradecane*, *pentadecane*, *hexadecane*, and *octadecane*), while the fraction collected at the bottom stream is the raffinate, that is, a phase rich in OLP or CO_2_, containing the less soluble compounds, in this case, the oxygenates (*palmitic acid*, *oleic acid*). 

**Figure 1:**
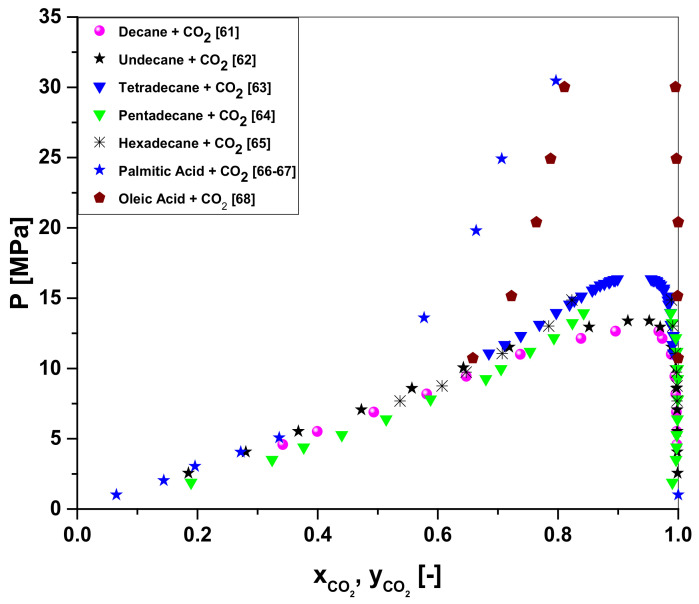
High-pressure phase equilibrium for the binary pairs OLP-compounds-i + CO_2_ (decane + CO_2_, undecane + CO_2_, tetradecane + CO_2_, pentadecane + CO_2_, hexadecane + CO_2_, palmitic acid + CO_2_, and oleic acid + CO_2_) described in the literature [61,62,63,64,65,66,67,68].

The P-x_CO2_,y_CO2_ diagram for the high-pressure phase equilibrium data for the binary pairs of OLP-compounds-i + CO_2_ (decane + CO_2_, undecane + CO_2_, tetradecane + CO_2_, pentadecane + CO_2_, hexadecane + CO_2_, palmitic acid + CO_2_, and oleic acid + CO_2_) described in the literature [61,62,63,64,65,66,67,68], shows that phase envelop of all the binary pairs of hydrocarbons-i + CO_2_ (decane + CO_2_, undecane + CO_2_, tetradecane + CO_2_, pentadecane + CO_2_, hexadecane + CO_2_) closes between 12.654 and 16.354 MPa and the phase envelope of binary pairs of hydrocarbons-i + CO_2_ limits the separation of hydrocarbons and carboxylic acids (oxygenates), as shown in Figure 1. In this context, the simulation should be performed between 12.654 and 16.354 MPa. In fact, the state conditions (P, T) to be chosen should take into account not only the solubility of OLP in the SC-CO_2_, a physicochemical property associated with the solvent-to-feed ratio (S/F), but also the fraction of carboxylic acids into OLP dissolved in SC-CO_2_ (selectivity). The higher the solubility of OLP in the SC-CO_2_, the lower the solvent-to-feed ratio (S/F) and the lower the selectivity. On the other hand, the lower the solubility of OLP in the SC-CO_2_, the higher the solvent-to-feed ratio (S/F) and the higher the selectivity. Therefore, a compromise must be found between the solubility of OLP in the SC-CO_2_ and the selectivity of OLP compounds in SC-CO_2_, that is, between the solvent-to-feed ratio (S/F) and the fraction of carboxylic acids into OLP dissolved in SC-CO_2_, as the deoxygenation aims to obtain extracts, that is, OLP containing very low concentrations carboxylic acids and raffinates with high concentrations of carboxylic acids. On the basis of the analysis of high-pressure phase equilibrium data for the binary pairs OLP-compounds-i + CO_2_, the state conditions chosen were *p* = 14.0 MPa and 333 K ≤ T ≤ 350 K.

The OLP deoxygenation process, using SC-CO_2_ as a solvent, was simulated using Aspen-HYSYS 8.6 (Aspen One, 2015). The simulations were performed by selecting the multistage countercurrent absorber/stripping unit operation because of its similarities and/or theoretical thermodynamic fundamentals relating to the solubility of gas in liquids, as the phase equilibrium behaves like a gas–liquid coexisting phase, as described elsewhere [59,60]. Aspen-HYSYS 8.6 computes the countercurrent multistage absorber column unit’s operations by applying the inside-out algorithm to solve, simultaneously, the materials and energies balances that are stage-to-stage coupled with a rigorous thermodynamic model. The RK-Aspen EOS with the adjusted binary interaction parameters was selected as the fluid package to compute the mixture properties for the multicomponent mixture OLP/SC-CO_2_ [59,60]. 

Organic liquid products obtained through the thermal catalytic cracking of palm oil at 450 °C, 1.0 atmosphere, with 10% (wt.) Na_2_CO_3_ as a catalyst, containing 89.24% (area.) hydrocarbons and 10% (area.) oxygenates were used as the feed stream [9,59]. The operating conditions in the simulation of the OLP deoxygenation process, using SC-CO_2_ as a solvent, with the help of the chemical process simulator Aspen-HYSYS 8.6 (Aspen One, 2015), are described in Table 1.

The process performance was evaluated on the basis of the yields of hydrocarbons and oxygenates, as well as the recovery of hydrocarbons, olefins, oxygenates, and carboxylic acid in the OLP fraction process streams. The yield and recovery of process stream i are computed as follows:

(1) $$Yield_{i}\left[\%\right]=\frac{\dot{m_{i}}}{{\dot{m}}_{OLP}}$$

(2) $$Recovery_{j}\left[\%\right]=\frac{\omega_{j*}\dot{m_{i}}}{\omega_{j,OLP*}{\dot{m}}_{OLP}}$$

where ${\dot{m}}_{i}$ and ${\dot{m}}_{OLP}$ are the mass flow rates of process stream *i* and the OLP feed stream, and $\omega_{j}$ and $\omega_{j,OLP}$ are the mass fractions of hydrocarbons or oxygenates chemical functions of process stream *i* and the OLP feed stream.

### 2.2. Simulation Methodology and Procedures

The Aspen-HYSYS chemical process simulator uses, for all separation processes and procedures, rigorous calculations for the mass and energy balances, providing a strong basis for simulations involving unit operations, thermodynamics, and chemical reactors. The software has a huge amount of flexibility and can simulate different chemical processes according to the user’s assembly [59].

**Figure 2:**
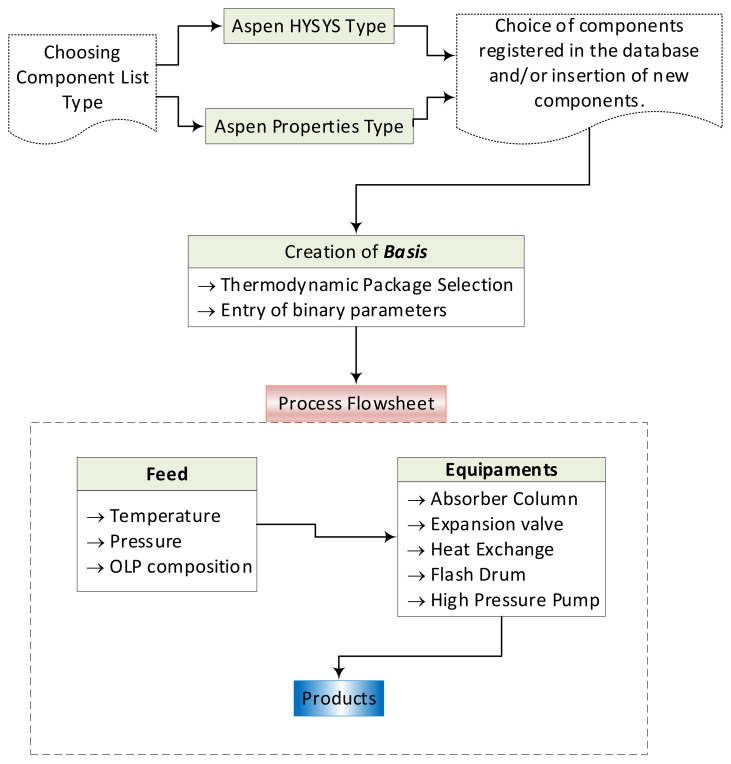
Process simulation steps using Aspen-HYSYS 8.4 for the deacidification/deoxygenation of OLP, as described elsewhere [59]: 1—the selection of the component list type; 2—the formulation of the list of components; 3—the basis formulation; 4—the flowsheet assembly of the process.

In this work, Aspen-HYSYS was used to perform simulations aiming to deacidify OLP in a multistage absorber column, in countercurrent mode, using SC-CO_2_ as a solvent. In general, process simulations using Aspen-HYSYS 8.4 consist of in the following steps [59]: 1—the selection of the component list type; 2—the formulation of the list of components; 3—the basis formulation; 4—the flowsheet assembly of the process, as shown in Figure 2.

### 2.3. Simulation Process Flowsheet Strategy

In a previous work, Costa et al. [60] provided the thermodynamic data basis and the EOS modeling to simulate the deoxygenation of OLP obtained through the thermal-catalytic cracking of palm oil at 450 °C, 1.0 atm, with 10% (wt.) Na_2_CO_3_ [9], making it possible to perform the process simulation of OLP deoxygenation with a multistage countercurrent absorber with SC-CO_2_ as a solvent using the process simulator Aspen-HYSYS. Costa et al. [59], simulated the fractionation of OLP obtained through the thermal catalytic cracking of palm oil at 450 °C, 1.0 atm, with 10% (wt.) Na_2_CO_3_ in countercurrent multistage absorber columns using CO_2_ as a solvent with Aspen-HYSYS, proposing flowsheets with 01 (one) absorber column (flowsheet I) and 02 (two) absorber columns (flowsheet II), where the bottom stream of first absorber column was used as a feed in the second absorber column using SC-CO_2_ as solvent, as described in detail in the literature [59]. This work addresses a new flowsheet, consisting of 03 (three) absorber columns, aiming to improve the deacidification of organic liquid products. 

**Figure 3:**
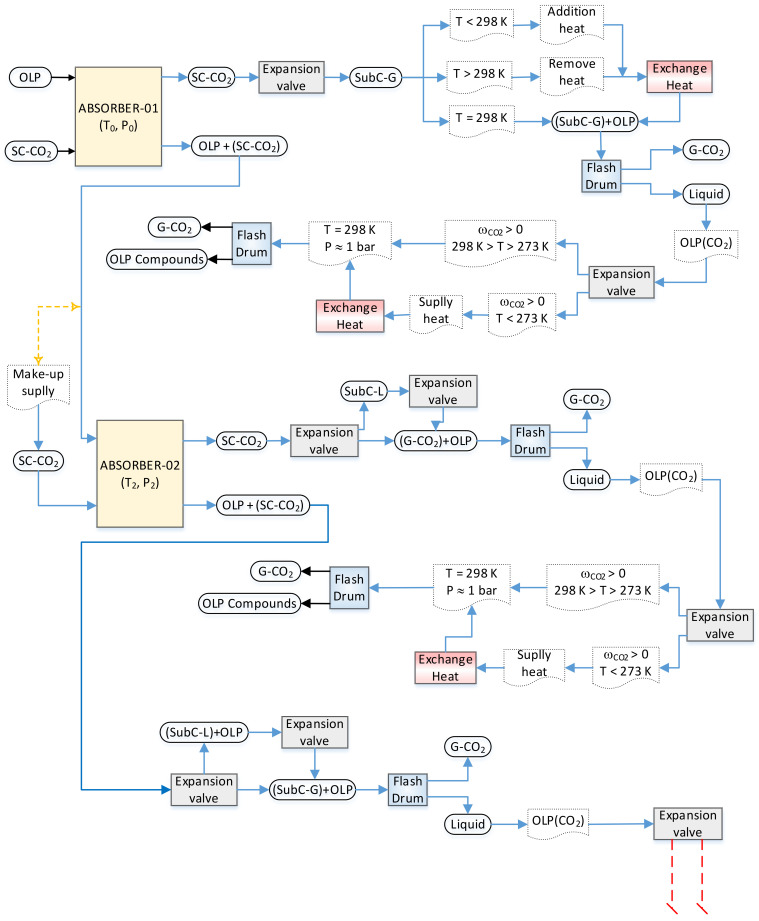
Flowsheet algorithm simulation strategy for the deacidification/deoxygenation of organic liquid products, obtained through the thermal catalytic cracking of palm oil at 450 °C, 1.0 atm, with 10% (wt.) Na_2_CO_3_ as a catalyst, in multistage countercurrent absorber columns using SC-CO_2_ as a solvent, illustrating sub-flowsheet I with absorbers 01 and 02.

**Figure 4:**
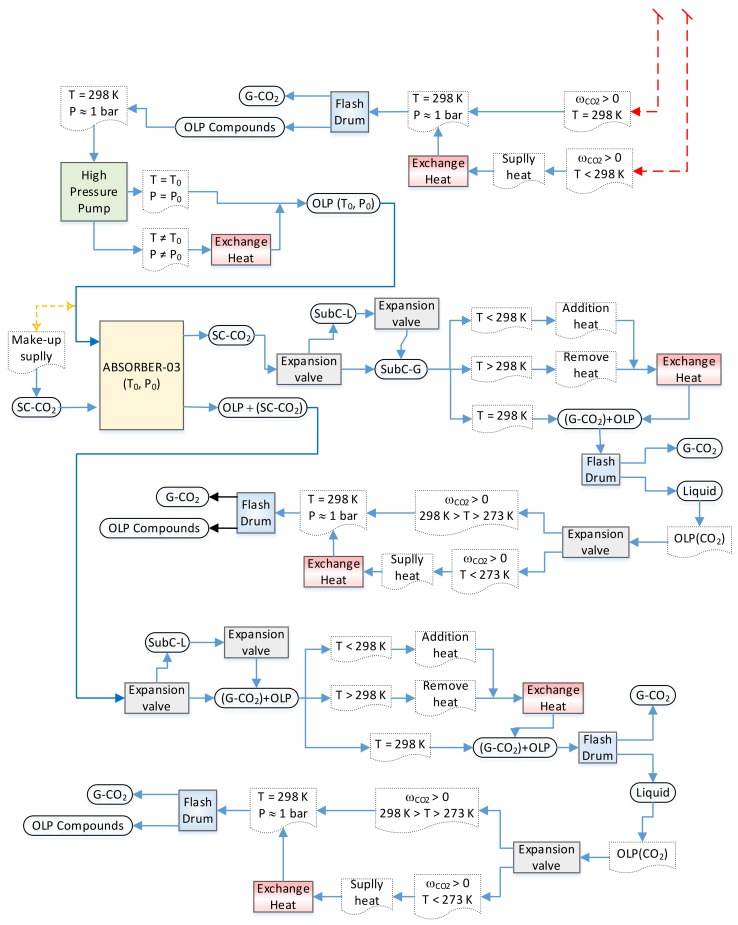
Flowsheet algorithm simulation strategy for the deacidification/deoxygenation of organic liquid products, obtained through the thermal catalytic cracking of palm oil at 450 °C, 1.0 atm, with 10% (wt.) Na_2_CO_3_ as a catalyst, in multistage countercurrent absorber columns using SC-CO_2_ as a solvent, illustrating sub-flowsheet II with absorber 03.

The algorithm, shown in the sub-flowsheets I and II illustrated in Figure 3 and Figure 4, respectively, describes in detail the procedures necessary to perform the simulation of OLP deacidification/deoxygenation, with the OLP obtained through the thermal catalytic cracking of palm oil at 450 °C, 1.0 atm, with 10% (wt.) Na_2_CO_3_ as a catalyst, in multistage countercurrent absorber columns using SC-CO_2_ as a solvent. Initially, OLP enters at the top of absorber-01 and SC-CO_2_ at the bottom in countercurrent mode. After equilibrium is achieved at a particular state condition (T_1_, P_1_), 02 (two) coexisting phases, including a dense gaseous phase, rich in SC-CO_2_ and containing the highly soluble/dissolved compounds (hydrocarbons) of OLP, and a liquid phase, poor in OLP, containing large amounts of dissolved CO_2_, are present. The dense gaseous phase exits the top of absorber-01, while the liquid phase exits the bottom of absorber-01. In order to remove CO_2_ in the top stream, it is necessary not only perform a state transition of SC-CO_2_ → G-CO_2_, but also guarantee that light OLP compounds are no longer dissolved in G-CO_2_. In this context, it is necessary to change the state conditions by adding an expansion valve followed by a heat exchanger to remove or add thermal energy, if necessary, to bring the temperature close to ambient conditions, as well as a flash drum to separate subcritical gaseous CO_2_ and/or gaseous CO_2_ from OLP. If the state conditions of the OLP that leaves the bottom of flash drum still permits dissolved CO_2_ to coexist in the OLP, then it is necessary to add an expansion valve to bring the pressure close to ambient conditions, followed by a heat exchanger to add thermal energy, if necessary, to bring the temperature close to ambient conditions (T = 298 K), as well as a flash drum to separate gaseous CO_2_ from the OLP. If all the gaseous CO_2_ is released at the top of the flash drum by ambient conditions (T ≈ 298 K, *p* ≈ 1.0 bar) and the OLP that leaves the bottom of the flash drum no longer contains dissolved CO_2_, then the separation step is completed.

The liquid that exits the bottom of absorber-01, poor in OLP, containing large amounts of dissolved CO_2_, enriched by the lowly soluble/dissolved compounds (oxygenates) of OLP enters at the top of absorber-02 as the feed, while fresh SC-CO_2_ (make-up) enters at the bottom of absorber-02 at the same state condition of the liquid stream (T_2_, P_1_). After equilibrium is achieved (T_2_, P_1_), 02 (two) coexisting phases, including a dense gaseous phase, rich in SC-CO_2_, containing the highly soluble/dissolved compounds (hydrocarbons) of OLP, and a liquid phase, poor in OLP, containing large amounts of dissolved CO_2_, are present. The dense gaseous phase exits the top of absorber-02, while the liquid phase exits the bottom of absorber-02. In order to remove CO_2_ in the top stream, it is necessary not only perform a state transition of SC-CO_2_ → G-CO_2_, but also guarantee that light OLP compounds are no longer dissolved in G-CO_2_. Adding an expansion valve makes it possible to change the state conditions, while a heat exchanger can be used to remove or add thermal energy, *if necessary*, to bring the temperature close to ambient conditions. Afterwards, a flash drum is added to separate the subcritical gaseous CO_2_ and/or gaseous CO_2_ from the OLP. If the OLP that leaves the bottom of the flash drum still contains dissolved CO_2_, then an expansion valve is added to bring the pressure close to ambient conditions, followed by a heat exchanger to supply thermal energy, *if necessary*, to bring the temperature close to ambient conditions (T = 298 K), as well as a flash drum to separate gaseous CO_2_ from the OLP. If all the gaseous CO_2_ is released at the top of the flash drum by ambient conditions (T ≈ 298 K, *p* ≈ 1.0 bar) and the OLP that leaves the bottom of the flash drum no longer contains dissolved CO_2_, then the separation step is completed.

The liquid phase leaving the bottom of absorber-02, poor in OLP, contains large amounts of dissolved CO_2_ that must be removed and separated from the OLP. In order to remove CO_2_ in the bottom stream, it is necessary to change the state conditions to diminish the solubility of the CO_2_ in the OLP. Adding an expansion valve makes it possible to change the state conditions of soluble/dissolved CO_2_ from liquid to subcritical gaseous CO_2_ and/or gaseous CO_2_ (L-CO_2_ → SC-CO_2_/G-CO_2_). Afterwards, a flash drum is added to separate the subcritical gaseous CO_2_ and/or gaseous CO_2_ from the OLP. If the OLP that leaves the bottom of the flash drum still contains dissolved CO_2_, then an expansion valve is added to bring the pressure close to ambient conditions, followed by a heat exchanger to supply thermal energy, *if necessary*, to bring the temperature close to ambient conditions (T = 298 K), as well as a flash drum to separate gaseous CO_2_ from the OLP. If all the gaseous CO_2_ is released at the top of the flash drum by ambient conditions (T ≈ 298 K, *p* ≈ 1.0 bar), but considerable amounts of OLP still leaves the bottom of the flash drum, even though it no longer contains dissolved CO_2_, another absorber should be added to the flowsheet. In order to bring the OLP to the state conditions (T_1_, P_1_) of absorber-03, a high-pressure pump is added after the flash drum bottom stream, followed by a heat exchanger to supply thermal energy. The OLP enters at the top of absorber-03 as the feed, while fresh SC-CO_2_ (make-up) enters at the bottom of absorber-03 at the same state condition of the liquid stream (T_1_, P_1_). After equilibrium is achieved (T_1_, P_1_), 02 (two) coexisting phases, including a dense gaseous phase, rich in SC-CO_2_, containing the highly soluble/dissolved compounds (hydrocarbons) of the OLP, and a liquid phase, poor in OLP, containing large amounts of dissolved CO_2_, are present. The dense gaseous phase exits the top of absorber-03, while the liquid phase exits the bottom of absorber-03. In order to remove CO_2_ in the top stream, it is necessary not only perform a state transition of SC-CO_2_ → G-CO_2_, but also guarantee that light OLP compounds are no longer dissolved in G-CO_2_. Adding an expansion valve makes it possible to change the state conditions, and a heat exchanger can be added to remove or add thermal energy, *if necessary*, to bring the temperature close to ambient conditions. Afterwards, a flash drum is added to separate the gaseous CO_2_ from the OLP. If the OLP that leaves the bottom of the flash drum still contains dissolved CO_2_, then an expansion valve is added to bring the pressure close to ambient conditions, followed by a heat exchanger to supply thermal energy, *if necessary*, to bring the temperature close to ambient conditions (T = 298 K), as well as a flash drum to separate gaseous CO_2_ from the OLP. If all the gaseous CO_2_ is released at the top of the flash drum by ambient conditions (T ≈ 298 K, *p* ≈ 1.0 bar) and the OLP that leaves the bottom of the flash drum no longer contains dissolved CO_2_, then the separation step is completed.

The liquid phase leaving the bottom of absorber-03, poor in OLP, contains large amounts of dissolved CO_2_ that must be removed and separated from OLP. In order to remove CO_2_ in the bottom stream, it is necessary to change the state conditions to diminish the solubility of the CO_2_ in the OLP. Adding an expansion valve makes it possible to change the state conditions of soluble/dissolved CO_2_ from liquid to gaseous CO_2_ (L-CO_2_ → G-CO_2_). Afterwards, a flash drum is added to separate the gaseous CO_2_ from the OLP. If the OLP that leaves the bottom of the flash drum still contains dissolved CO_2_, then an expansion valve is added to bring the pressure close to *p* ≈ 1.0 bar, followed by a heat exchanger to supply thermal energy, *if necessary*, to bring the temperature close to T ≈ 298 K, as well as a flash drum to separate gaseous CO_2_ from the OLP. If all the gaseous CO_2_ is released at the top of the flash drum by ambient conditions (T ≈ 298 K, *p* ≈ 1.0 bar) and the OLP that leaves the bottom of the flash drum no longer contains dissolved CO_2_, then the separation step is completed.

## 3. Results and Discussions

### 3.1. Process Simulation

#### Process Flowsheet

**Figure 5:**
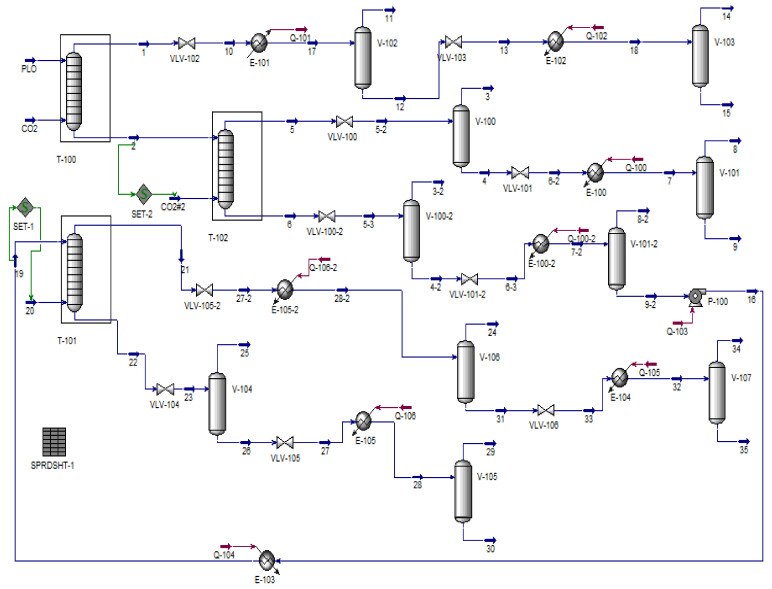
Process flowsheet (absorber columns (T-100, T-101, T-102), flash drums (V-100, V-100-2, V-101, V-101-2, V-102, V-103, V-104, V-105, V-106, V-107), valves (VLV-100, VLV-100-2, VLV-101, VLV-101-2, VLV-102, VLV-103, VLV-104, VLV-105, VLV-105-2, VLV-106), heat exchanges (E-100, E-100-2, E-101, E-102, E-103, E-104, E-105, E-105-2), make-ups (SET-1, SET-2), high-pressure pump (P-100)) for OLP deoxygenation in multistage countercurrent absorber columns using CO_2_ as a solvent (raffinate 1 + CO_2_ as the feed for the top stream of absorber T-102, raffinate 2 as the feed for the top stream of absorber T-101, and a CO_2_ make-up as the feed for the bottom stream of absorber T-102 and of absorber T-101).

The proposed flowsheet illustrated in Figure 5 consists of 03 absorber columns, 10 expansions valves, 10 flash drums, 08 heat exchanges, 01 high-pressure pump, and 02 make-ups of CO_2_, aiming to optimize the deacidification of organic liquid products.

Table 2 shows the material (molar and mass flow rates) and energy balances (heat flow rates), fractions of gas phases, as well as the state conditions (T, P) for all the process streams for the simulation of the OLP deoxygenation process in multistage countercurrent absorber columns using CO_2_ as a solvent, with Aspen-HYSYS 8.6 (Aspen One, 2015) as the simulator, expressed on a CO_2_ basis, at 333 K, 140 bar, and (S/F) = 17; at 350 K, 140 bar, and (S/F) = 25; and at 333 K, 140 bar, and (S/F) = 38.

In addition, Supplementary Tables S1–S3 provide detailed information on the process conditions, mass flow rates, gaseous fractions, recoveries of hydrocarbons and oxygenates in extract and raffinates, as well as the chemical composition, expressed on a solvent-free basis, of the hydrocarbons and oxygenates of OLP in the feed, top, and bottom streams of absorber columns T-100, T-102, and T-101 for the simulation of the OLP deoxygenation process in multistage countercurrent absorber columns using CO_2_ as a solvent, with Aspen-HYSYS 8.6 (Aspen One, 2015) as the simulator, at 333 K, 140 bar, and (S/F) = 17; at 350 K, 140 bar, and (S/F) = 25; and at 333 K, 140 bar, and (S/F) = 38.

Stream 1, leaving the top of absorber column T-100, rich in carbon dioxide in a supercritical state, with a gaseous phase solubility **y_CO2,OLP_** = 0.9597, which is very close to **y_CO2,Undecane_** = 0.9518 at 344.5 K and 13.4 MPa for the high-pressure phase equilibria of binary system n-undecane + CO_2_ [62], flows through the expansion valve (VLV-102), causing not only a decrease in pressure and temperature, but also a phase change from a supercritical to a sub-critical gaseous state, making it possible to separate CO_2_ in the gaseous state from organic liquid products. However, if the solubility of dissolved the OLP compounds in gaseous CO_2_ is still low but not zero, due to high temperatures at the exit of expansion valve, a heat exchanger (E-101) is placed after the expansion valve to diminish the temperature, thus making it possible to complete the separation of gaseous CO_2_ and the dissolved OLP compounds within the flash drum (V-102), since the solubility of dissolved OLP in CO_2_ tends to zero. The bottom stream of the flash drum (V-102), rich in OLP compounds, still contains dissolved CO_2_, thus making it necessary to insert another expansion valve (VLV-103) at the exit of the flash drum (V-102), causing a decrease in pressure and temperature. However, if the temperature is too low, then that may solidify the condensed OLP compounds, and so a second heat exchanger (E-102) should be placed after the expansion valve to supply thermal energy in order to increase the temperature, maintaining the OLP compounds in the liquid state, thus making it possible to complete the separation of gaseous CO_2_ and the condensed OLP compounds within the flash drum (V-103), since the solubility of dissolved CO_2_ in OLP tends to zero. 

The OLP at the top stream of absorber column T-100, expressed on a solvent-free basis, obtained from bottom stream 15, after the separation and/or degassing of gaseous CO_2_ in the flash drums V-102 and V-103, present as their chemical composition 96.95% (area.) hydrocarbons with 39.14% (area.) alkanes, 36.39% (area.) alkenes (olefins), and 21.42% (area.) naphthene, as well as 3.05% (area.) oxygenates. By comparing the composition of the OLP in stream 15 with that of a kerosene-like fraction, containing 93.63% (area.) hydrocarbons (42.62% alkanes, 24.89% alkenes, and 26.12% naphthene) and 6.37% (area.) oxygenates, obtained through the distillation of organic liquid products after the thermal catalytic cracking of dehydrated residual fat, oils, and grease (FOG) from grease traps at 450 °C, 1.0 atm, with 10% (wt.) Na_2_CO_3_, at a pilot scale [19], we see that the OLP fraction of the extract in bottom stream 15 of absorber column T-100 resembles the chemical composition of kerosene, with lower levels of alkanes and oxygenates. In addition, the recovery of OLP, expressed on a solvent-free basis, obtained in bottom stream 15 was 36.65%, as shown in Table 3.

Stream 2, leaving the bottom of absorber column T-100, rich in CO_2_ dissolved in OLP, with a liquid phase solubility **x_CO2,OLP_** = 0.9281, which is very close to **x_CO2,Undecane_** = 0.9161 at 344.5 K and 13.4 MPa for the high-pressure phase equilibria of binary system n-undecane + CO_2_ [62], enters the top of absorber column T-102 as partially acidified hydrocarbons, containing 84.78% (area.) hydrocarbons (43.54% alkanes, 18.95% alkenes, and 22.29% naphthene) and 15.22% (area.) oxygenates. Fresh CO_2_, supplied as a make-up gas, enters the bottom of absorber column T-102 in order to achieve the solvent-to-feed ratio of (S/F) = 38. Stream 5, leaving the top of absorber column T-102, rich in carbon dioxide in the supercritical state, with a gaseous phase solubility **y_CO2,OLP_** = 0.9817, which is very close to **y_CO2,Tetradecane_** = 0.9870 at 344.5 K and 13.95 MPa for the high-pressure phase equilibria of binary system n-undecane + CO_2_ [62], flows through the expansion valve (VLV-100), changing the state conditions (T = 294.6 K, *p* = 40 bar), causing a phase change from a supercritical to a gaseous state, making it possible to separate CO_2_ in the gaseous state is there the reference citationfrom organic liquid products inside the flash drum (V-100). The bottom stream of the flash drum (V-100), rich in OLP compounds, still contains dissolved CO_2_, thus making it necessary to insert another expansion valve (VLV-101) at the exit of the flash drum (V-100), causing a decrease in pressure and temperature (T = 236.0 K, *p* = 1.5 bar). In cases where a drastic decrease in the temperature takes place because of the Joule–Thomson effect, which may solidify the OLP compounds, a heat exchanger (E-100) is placed after the expansion valve to supply thermal energy to bring the temperature to ambient conditions (T = 298 K), thus making it possible to complete the separation of gaseous CO_2_ and the dissolved OLP compounds within the flash drum (V-101), since the solubility of dissolved OLP in CO_2_ tends to zero.

The OLP at the top stream of absorber column T-102, expressed on a solvent-free basis, obtained from bottom stream 9, after the separation and/or degassing of gaseous CO_2_ in the flash drums V-100 and V-101, contains 92.78% (area.) hydrocarbons (46.53% alkanes, 21.75% alkenes, and 24.49% naphthene) and 7.22% (area.) oxygenates. By comparing the composition of OLP in stream 9 with that of a gasoline-like fraction containing 92.30% (area.) hydrocarbons (21.52% alkanes, 37.51% alkenes, and 33.27% naphthene) and 7.70% (area.) oxygenates, obtained through the distillation of organic liquid products after the thermal catalytic cracking of palm oil at 450 °C, 1.0 atm, with 20% (wt.) Na_2_CO_3_, at a pilot scale [69], we see that the OLP fraction of the extract in bottom stream 9 of absorber column T-102 resembles the chemical composition of gasoline, with lower levels of alkanes and oxygenates. In addition, the recovery of OLP, expressed on a solvent-free basis, obtained from bottom stream 9 was 40.77%, as shown in Table 3.

Stream 6, leaving the bottom of absorber column T-102, rich in dissolved CO_2_ into OLP, with a liquid phase solubility **x_CO2,OLP_** = 0.9072, which is very close to **x_CO2,Undecane_** = 0.9161 at 344.5 K and 13.4 MPa for the high-pressure phase equilibria of binary system n-undecane + CO_2_ [62], flows through the expansion valve (VLV-100-2), changing the state conditions, causing a phase change from a supercritical (T = 371 K, *p* = 140 bar) to a sub-critical gaseous state (T = 319 K, *p* = 50 bar), making it possible to separate CO_2_ in the gaseous state from organic liquid products inside the flash drum (V-100-2). The bottom stream of the flash drum (V-100-2), rich in OLP compounds, still contains dissolved CO_2_, thus making it necessary to insert another expansion valve (VLV-101-2) at the exit of the flash drum (V-100-2), causing a decrease in pressure (*p* = 1.5 bar) and temperature (T = 291 K). In order to bring the temperature to ambient conditions, a heat exchanger (E-100-2) is placed after the expansion valve to supply thermal energy, thus making it possible to complete the separation of gaseous CO_2_ and the dissolved OLP compounds within the flash drum (V-101-2), since the solubility of dissolved OLP in CO_2_ tends to zero. As the stream leaving the bottom of the flash drum (V-101-2) still contains large amounts of OLP compounds, a third absorber column is added to the flowsheet to deacidify the OLP compounds. Stream 9-2 is compressed with the help of a high-pressure pump (P-100) to bring the OLP to the state conditions of absorber column T-101 (T = 333 K, *p* = 140 bar). After the high-pressure pump, the state condition of stream 16 is 303 K and 140 bar, thus making it necessary to add a heat exchanger (E-103) to supply thermal energy to bring the temperature to 333 K. Stream 19 (T = 333 K, *p* = 140 bar) enters the top of absorber column T-101, while fresh CO_2_ supplied as a make-up gas (SET-1) enters the bottom of absorber column T-101 at 333 K and 140 bar.

Stream 21, leaving the top of absorber column T-101, rich in CO_2_ in the supercritical state, with a gaseous phase solubility **y_CO2,OLP_** = 0.9869, which is very close to **y_CO2,Tetradecane_** = 0.9870 at 344.5 K and 13.95 MPa for the high-pressure phase equilibria of binary system n-undecane + CO_2_ [63], flows through the expansion valve (VLV-105-2), causing a phase change from a supercritical to a gaseous state, making it possible to separate CO_2_ in the gaseous state from organic liquid products. If the temperature is lower than 298 K, a heat exchanger (E-105-2) is placed after the expansion valve to bring the temperature to 298 K, thus making the separation of gaseous CO_2_ and the dissolved OLP compounds within the flash drum possible (V-106). If the bottom stream of the flash drum (V-106), rich in OLP compounds, still contains dissolved CO_2_, it is necessary to insert another expansion valve (VLV-106) at the exit of the flash drum (V-106), causing a decrease in pressure and temperature. However, if the temperature is too low, then that may solidify the condensed OLP compounds, and a second heat exchanger (E-104) should be placed after the expansion valve (VLV-106) to supply thermal energy, in order to increase the temperature, maintaining the OLP compounds in the liquid state, thus making it possible to complete the separation of gaseous CO_2_ and the condensed OLP compounds within the flash drum (V-107), since the solubility of dissolved CO_2_ in OLP tends to zero. 

Stream 22, leaving the bottom of absorber column T-101, rich in dissolved CO_2_ into OLP, with a liquid phase solubility **x_CO2,OLP_** = 0.9323, which is very close to **x_CO2,Undecane_** = 0.9161 at 344.5 K and 13.4 MPa for the high-pressure phase equilibria of binary system n-undecane + CO_2_ [62], flows through the expansion valve (VLV-104), causing a phase change from a supercritical (T = 333 K, *p* = 140.0 bar) to a gaseous state (T = 298 K, *p* = 60 bar), making it possible to separate CO_2_ in the gaseous state from organic liquid products inside the flash drum (V-104). The bottom stream of the flash drum (V-104), rich in OLP compounds, still contains dissolved CO_2_, thus making it necessary to insert another expansion valve (VLV-105) at the exit of the flash drum (V-104), causing a decrease in pressure (*p* = 1.5 bar) and temperature (T = 191.0 K). In order to bring the temperature to ambient conditions, a heat exchanger (E-105) is placed after the expansion valve to supply thermal energy, thus making it possible to complete the separation of gaseous CO_2_ and the dissolved OLP compounds within the flash drum (V-105), since the solubility of dissolved OLP in CO_2_ tends to zero at 298 K and 1.5 bar. The simulated gas–liquid high-pressure equilibrium in the exit of absorber columns T-100, T-102, and T-101, given by the **y**_CO2,OLP_ and **x**_CO2,OLP_ of streams 1, 2, 3, 6, 21, and 22, are defined according to high-pressure equilibrium data for the binary pairs of OLP-compounds-i + CO_2_ (*decane + CO_2_, undecane + CO_2_, tetradecane + CO_2_, pentadecane + CO_2_, hexadecane + CO_2_*) described in the literature [61,62,63,64,65].

The OLP at the bottom stream of absorber column T-102, expressed on a solvent-free basis, obtained from bottom stream 9-2, after the separation and/or degassing of gaseous CO_2_ in the flash drums V-100-2 and V-101-2, enriched on oxygenates, present as their composition 70.34% (area.) hydrocarbons (38.15% alkanes, 13.88% alkenes, and 18.31% naphthene) and 29.66% (area.) oxygenates, while stream 35 at the bottom flash drum (V-107), expressed on a solvent-free basis, contains 83.85% (area.) hydrocarbons and 16.15% (area.) oxygenates. Stream 30 at the bottom flash drum (V-105), expressed on a solvent-free basis, enriched in oxygenates, contains 67.34% (area.) hydrocarbons and 32.66% (area.) oxygenates. The recovery of OLP, expressed on a solvent-free basis, obtained from bottom stream 35 of the flash drum (V-107) was 4.07%, as shown in Table 3.

The simulated chemical composition of OLP in stream 15, expressed on a CO_2_-free basis, shows a yield of 36.65% with 96.95% (wt.) hydrocarbons and 3.05% (wt.) oxygenates. A decrease in the oxygenate content in feed OLP could be observed from 10% (wt.), with 2.63% (wt.) carboxylic acids, to 3.05% (wt.) oxygenates, with 0.52% (wt.) carboxylic acids [59], showing that the fraction of carboxylic acids in OLP dissolved into SC-CO_2_ was very low. This is according to high-pressure phase equilibrium data for the binary systems of palmitic acid + CO_2_ [66,67], and oleic acid + CO_2_ [68].

The simulated chemical composition of OLP in stream 9, expressed on a CO_2_-free basis, shows a yield of 40.77% with 92.78% (wt.) hydrocarbons and 7.22% (wt.) oxygenates. A decrease in the oxygenate content in RAF1 could be observed from 15.22% (wt.), with 3.85% (wt.) carboxylic acids, to 7.22% (wt.) oxygenates, with 1.47% (wt.) carboxylic acids [59], showing that the fraction of carboxylic acids in OLP dissolved into SC-CO_2_ was very low. This is according to high-pressure phase equilibrium data for the binary systems of palmitic acid + CO_2_ [66,67] and oleic acid + CO_2_ [68]. Finally, it can be observed in Table 3 that 81.49% (wt.) of feed OLP was recovered in the top streams of absorbers T-100, T-102, and T-101, showing that deacidified OLP have chemical compositions similar to kerosene-like fractions obtained through the distillation of organic liquid products after the thermal catalytic cracking of dehydrated residual fat, oils, and grease (FOG) from grease traps at 450 °C, 1.0 atm, with 10% (wt.) Na_2_CO_3_, at a pilot scale [19], and gasoline-like fractions obtained through the distillation of organic liquid products after the thermal catalytic cracking of palm oil at 450 °C, 1.0 atm, with 20% (wt.) Na_2_CO_3_, at a pilot scale [69].

## 4. Conclusions

This work addresses a new flowsheet, consisting of three absorber columns, aiming to improve the deacidification of organic liquid products (OLP) in multistage countercurrent absorber/stripping columns using SC-CO_2_ as a solvent, with the Aspen-Hysys system.

The algorithm, shown in sub-flowsheets I and II, describes in detail the procedures necessary to perform the simulation of OLP deacidification/deoxygenation, obtained through the thermal catalytic cracking of palm oil at 450 °C, 1.0 atm, with 10% (wt.) Na_2_CO_3_ as a catalyst, in multistage countercurrent absorber columns using SC-CO_2_ as a solvent.

The simulation shows that 81.49% of OLP could be recovered and that the concentrations of hydrocarbons in the extracts of absorber-01 and absorber-02 were 96.95 and 92.78% (wt.) on a solvent-free basis, while the bottom stream of absorber-03 was enriched in oxygenate compounds with concentrations up to 32.66% (wt.) on a solvent-free basis, showing that organic liquid products (OLP) was deacidified and SC-CO_2_ was able to deacidify OLP and obtain fractions with lower olefin contents. 

## Figures and Tables

**Table 1:** Operating conditions of the simulation of the OLP deoxygenation process, using SC-CO_2_ as solvent, with the help of the chemical process simulator Aspen-HYSYS 8.6 (Aspen One, 2015).

Operating Conditions	
Absorber Column 1	T = 333 K
Feed = 100 kg/h	*p* = 140 bar
Numbers of stages = 10	(S/F) = 17
Absorber Column 2	T = 350 K
Numbers of stages = 10	*p* = 140 bar
Feed = Raffinate 1	(S/F) = 38
Absorber Column 3	T = 333 K
Numbers of stages = 10	*p* = 140 bar
Feed = Raffinate 2	(S/F) = 25

**Table 2:** Material and energy balances and state conditions (T, P) for all the process streams for the simulation of the OLP deoxygenation process in multistage countercurrent absorber columns using CO_2_ as a solvent, with Aspen-HYSYS 8.6 (Aspen One, 2015) as the simulator, expressed on a CO_2_ basis, at 333 K, 140 bar, and (S/F) = 17; at 350 K, 140 bar, and (S/F) = 25; and at 333 K, 140 bar, and (S/F) = 38.

Stream Nº	ω_i,Gas_	T (°C)	*p* (Bar)	Molar Flow (kmol/h)	Mass Flow (kg/h)	Liquid Vol. Flow (m³/h)	Heat Flow (kcal/h)		Heat Flow (kcal/h)
PLO	0	60	140	0.5196	100	0.1214	−43,708.4	Q-100	1361.1
CO_2_	1	60	140	38.4232	1691	2.0578	−3,672,925.4	Q-101	19,905.9
1	1	147.94	140	20.0574	910.0584	1.1081	−1,872,017.7	Q-102	763.3
2	0.92522	77.78	140	18.8854	880.9416	1.0712	−1,794,358.8	Q-100-2	80.7
5	1	93.54	140	49.8952	2227.474	2.7105	−4,718,556.9	Q-103	154.0
6	0.93017	98.15	140	5.1121	243.186	0.2952	−483,682.8	Q-104	318.3
CO2#2	1	77.78	140	36.1219	1589.718	1.9346	−3,431,362.5	Q-106	2496.4
3	1	21.64	40	49.1959	2165.239	2.6349	−4,652,389.5	Q-106-2	1843.5
4	0	21.64	40	0.6993	62.2349	7.56 × 10^−3^	−66,167.4	Q-105	214.0
5-2	0.98598	21.64	40	49.8952	2227.474	2.7105	−4,718,556.9		
6-2	0.66532	−37.00	1.5	0.6993	62.2349	7.56 × 10^−2^	−66,167.4		
7	0.69432	25	1.5	0.6993	62.2349	7.56 × 10^−2^	−64,806.3		
8	1	25	1.5	0.4855	21.3767	2.60 × 10^−2^	−45,671.2		
9	0	25	1.5	0.213767	40.8581	4.95 × 10^−2^	−19,135.1		
10	0.98826	107.40	45	20.0574	910.0584	1.1081	−1,872,017.7		
11	1	35	45	19.4630	856.909	1.0428	−1,838,423.0		
12	0	35	45	0.5943	53.1494	6.53 × 10^−2^	−53,500.6		
13	0.62478	−14.02	1.5	0.5943	53.1494	6.53 × 10^−2^	−53,500.6		
14	1	25	1.5	0.3794	16.7190	2.03 × 10^−2^	−35,684.9		
15	0	25	1.5	0.2149	36.4304	4.49 × 10^−2^	−17,052.4		
17	0.97036	35	45	20.0574	910.0584	1.1081	−1,891,923.6		
18	0.63841	25	1.5	0.5943	53.1494	6.53 × 10^−2^	−52,737.3		
3-2	1	46.09	50	4.8689	214.2947	0.2607	−459,529.9		
4-2	0	46.09	50	0.2432	28.8913	3.45 × 10^−2^	−24,152.8		
5-3	0.95242	46.09	50	5.1121	243.186	0.2952	−483,682.8		
6-3	0.57602	18.04	1.5	0.2432	28.8913	3.45 × 10^−2^	−24,152.8		
7-2	0.57774	25	1.5	0.2432	28.8913	3.45 × 10^−2^	−24,072.1		
8-2	1	25	1.5	0.1405	6.1840	7.53 × 10^−3^	−13,217.3		
9-2	0	25	1.5	0.1026	22.7073	2.70 × 10^−2^	−10,854.8		
16	0	30.12	140	0.1026	22.7073	2.70 × 10^−2^	−10,700.7		
19	0	60	140	0.1026	22.7073	2.70 × 10^−2^	−10,382.4		
20	1	60	140	6.1480	270.5742	3.29 × 10^−1^	−587,699.0		
21	1	72.81	140	4.1244	185.1077	2.25 × 10^−1^	−392,745.7		
22	0.99080	60.39	140	2.1263	108.1738	0.1310	−204,486.5		
23	0.51057	25.12	60	2.1263	108.1738	0.1310	−204,486.5		
25	1	25.12	60	1.085	47.7824	5.81 × 10^−2^	−103,080.1		
29	1	25	1.5	0.9589	42.2025	5.14 × 10^−2^	−90,200.8		
30	0	25	1.5	8.18 × 10^−2^	18.1888	2.16 × 10^−2^	−8709.2		
27	0.68209	−82.52	1.5	1.0406	60.3913	7.29 × 10^−2^	−101,406.4		
28	0.92143	25	1.5	1.0406	60.3913	7.29 × 10^−2^	−98,910.0		
26	0	25.12	60	1.0406	60.3913	7.29 × 10^−2^	−101,406.4		
27-2	0.84043	15.85	50	4.1244	185.1077	2.25 × 10^−1^	−392,745.7		
28-2	0.97521	25	50	4.1244	185.1077	2.25 × 10^−1^	−390,902.2		
24	1	25	50	4.0221	177.0208	2.15 × 10^−1^	−380,896.7		
31	0	25	50	0.1022	8.0869	9.73 × 10^−3^	−10,005.5		
32	0.79566	25	1.5	0.1022	8.0869	9.73 × 10^−3^	−9791.4		
34	1	25	1.5	8.13 × 10^−2^	3.5796	4.36 × 10^−3^	−7650.9		
35	0	25	1.5	2.09 × 10^−2^	4.5072	5.37 × 10^−3^	−2140.5		
33	0.74861	−54.91	1.5	0.1022	8.0869	9.73 × 10^−3^	−10,005.5		

**Table 3:** Chemical composition, expressed on a solvent-free basis, of hydrocarbons and oxygenates of OLP in feed, top, and bottom streams of absorber columns T-100, T-102, and T-101 following the simulation of the OLP deoxygenation process in multistage countercurrent absorber columns using CO_2_ as a solvent, with Aspen-HYSYS 8.6 (Aspen One, 2015) as the simulator, expressed on a CO_2_ basis, at 333 K, 140 bar, and (S/F) = 17; at 350 K, 140 bar, and (S/F) = 25; and at 333 K, 140 bar, and (S/F) = 38.

	Feed	Column 1		Column 2		Column 3	
(S/F)	-	17		38		25	
	OLP	Top	Bottom (RAF1)	Top	Bottom (RAF2)	Top	Bottom
Mass Flow (kg/h)	100	36.65	63.35	40.77	22.58	4.07	18.49
Mass fraction (CO_2_-free basis)							
Hydrocarbons	0.8924	0.9695	0.8478	0.9278	0.7034	0.8385	0.6734
Alkanes	0.4193	0.3914	0.4354	0.4653	0.3815	0.4321	0.3701
Alkenes	0.2534	0.3639	0.1895	0.2175	0.1388	0.1389	0.1389
Naphthenes	0.2197	0.2142	0.2229	0.2449	0.1831	0.2674	0.1644
Oxygenates	0.1076	0.0305	0.1522	0.0722	0.2966	0.1615	0.3266
Carboxylic acids	0.0263	0.0052	0.0385	0.0147	0.0814	0.0354	0.0916
Alcohols	0.0351	0.0086	0.0505	0.0174	0.1102	0.0412	0.1255
Ketones	0.0462	0.0167	0.0632	0.0402	0.1049	0.0849	0.1094

## Supplementary Materials

The following supporting information can be downloaded at: https://www.mdpi.com/article/10.3390/molecules27072211/s1. Table S1: Process conditions, mass flow rates, gaseous fractions, and recoveries of hydrocarbons and oxygenates in extracts and raffinates, as well as chemical composition, expressed on a solvent-free basis, of hydrocarbons and oxygenates of OLP in feed, top, and bottom streams of absorber columns T-100 for the simulation of the OLP deoxygenation process in multistage countercurrent absorber columns using CO_2_ as a solvent, with Aspen-HYSYS 8.6 (Aspen One, 2015) as the simulator, at 333 K, 140 bar, and (S/F) = 17. Table S2: Process conditions, mass flow rates, gaseous fractions, and recoveries of hydrocarbons and oxygenates in extracts and raffinates, as well as chemical composition, expressed on a solvent-free basis, of hydrocarbons and oxygenates of OLP in feed, top, and bottom streams of absorber columns T-102 for the simulation of the OLP deoxygenation process in multistage countercurrent absorber columns using CO_2_ as a solvent, with Aspen-HYSYS 8.6 (Aspen One, 2015) as the simulator, at 350 K, 140 bar, and (S/F) = 25. Table S3: Process conditions, mass flow rates, gaseous fractions, and recoveries of hydrocarbons and oxygenates in extracts and raffinates, as well as chemical composition, expressed on a solvent-free basis, of hydrocarbons and oxygenates of OLP in feed, top, and bottom streams of absorber columns T-101 for the simulation of the OLP deoxygenation process in multistage countercurrent absorber columns using CO_2_ as a solvent, with Aspen-HYSYS 8.6 (Aspen One, 2015) as the simulator, at 333 K, 140 bar, and (S/F) = 38.

## Sample Availability

Not available.
