# Supplementary material for: Simulation of Organic Liquid Product Deoxygenation through Multistage Countercurrent Absorber/Stripping Using CO2 as Solvent with Aspen-HYSYS: Process Modeling and Simulation
Source: Molecules. 2022 Mar 29;27(7):2211. doi: 10.3390/molecules27072211 (PMC9000492; doi:10.3390/molecules27072211)
Supplement: Supplementary file 1 [file molecules-27-02211-s001.zip › molecules-1558224-supplementary.pdf]

ABSORBER T-100, (S/F) = 17					CO_2 (Free-Basis)	CO_2 (Free-Basis)
Streams	**PLO**	**CO_2**	**1**	**2**	**Extract**	**Raffinate**
Gas fraction	0.0000	1.0000	1.0000	0.0000		
**Temperature [C]**	**60.0**	60.0	148.0	80.1		
**Pressure [bar]**	**140**	140	140	140		
Molar Flow [kgmole/h]	0.52	38.42	20.06	18.89		
Mass Flow [kg/h]	100.00	1691.00	910.06	880.94	**36.65**	**63.35**
**Composition of OLP (Mass Fraction)**						
Carbon-Dioxide	0.0000	1	0.9597	0.9281	0.0000	0.0000
n-Undecane	0.0641	0	0.0046	0.0026	0.1130	0.0358
n-Dodecane	0.0315	0	0.0020	0.0016	0.0485	0.0216
n-Tridecane	0.0526	0	0.0025	0.0034	0.0627	0.0467
n-Tetradecane	0.0925	0	0.0029	0.0075	0.0724	0.1041
n-Pentadecane	0.1259	0	0.0028	0.0114	0.0705	0.1580
n-Hexadecane	0.0274	0	0.0007	0.0024	0.0171	0.0333
n-Heptadecane	0.0132	0	0.0002	0.0013	0.0055	0.0177
n-Octadecane	0.0121	0	0.0001	0.0013	0.0017	0.0181
1-Decene	0.0469	0	0.0047	0.0005	0.1157	0.0071
cis-2-Decene	0.0047	0	0.0005	0.0001	0.0113	0.0009
1-Dodecene	0.0985	0	0.0064	0.0046	0.1583	0.0639
trans-2-Dodecene	0.0038	0	0.0002	0.0002	0.0057	0.0027
1-Tridecene	0.0019	0	0.0001	0.0001	0.0024	0.0016
1-Tetradecene	0.0052	0	0.0002	0.0004	0.0050	0.0053
(E)-9-Octadecene	0.0061	0	0.0001	0.0006	0.0018	0.0086
1-Pentadecene	0.0043	0	0.0001	0.0004	0.0031	0.0050
1-Heptadecene	0.0331	0	0.0006	0.0032	0.0142	0.0440
1-Nonadecene	0.0082	0	0.0001	0.0008	0.0021	0.0117
1,4-Undecadiene	0.0056	0	0.0005	0.0002	0.0113	0.0023
5-Icosene	0.0008	0	0.0000	0.0001	0.0002	0.0011
Isobutylcyclohexane	0.0059	0	0.0006	0.0001	0.0145	0.0009
n-Butylcyclohexane	0.0038	0	0.0003	0.0001	0.0087	0.0010
Cyclododecane	0.0048	0	0.0002	0.0003	0.0051	0.0046
n-Decylcyclopentane	0.0594	0	0.0015	0.0052	0.0364	0.0727
n-Nonylcyclopentane	0.0364	0	0.0012	0.0029	0.0293	0.0405
n-Nonylcyclohexane	0.0517	0	0.0012	0.0046	0.0307	0.0638
1-Ethyl-2 heptylcyclopropane	0.0048	0	0.0003	0.0002	0.0076	0.0032
1-Pentyl-2-propylcyclopropane	0.0197	0	0.0016	0.0006	0.0389	0.0086
1-Butyl-1-cyclohexene	0.0072	0	0.0007	0.0001	0.0165	0.0018
1-Hexylcyclopentene	0.0057	0	0.0004	0.0002	0.0101	0.0031
6-Dodecyne	0.0109	0	0.0007	0.0005	0.0176	0.0070
1-Heptyl-1-cyclohexene	0.0032	0	0.0001	0.0002	0.0037	0.0029
o-Cresol	0.0014	0	0.0001	0.0000	0.0029	0.0005
9-Octadecen-1-ol,-(Z)-	0.0337	0	0.0002	0.0036	0.0057	0.0499
2-Pentadecanone	0.0462	0	0.0007	0.0045	0.0167	0.0632
Palmitic acid	0.0175	0	0.0002	0.0018	0.0044	0.0251
Oleic acid	0.0088	0	0.0000	0.0010	0.0008	0.0134
1-Decylcyclohexene	0.0027	0	0.0001	0.0002	0.0017	0.0033
3-Hexadecene	0.0234	0	0.0006	0.0020	0.0151	0.0282
1-Nonil-CicloHexene	0.0144	0	0.0004	0.0012	0.0109	0.0164
**Composition of OLP in Feed (Mass Fraction)**					**Composition of OLP in Extract (Mass Fraction)**	**Composition of OLP in Raffinate (Mass Fraction)**
**Hydrocarbons**	**0.8924**	**0.0000**	**0.0390**	**0.0610**	**0.9695**	**0.8478**
Alkanes	0.4193	0.0000	0.0158	0.0313	0.3914	0.4354
Alkenes	0.2534	0.0000	0.0147	0.0136	0.3639	0.1895
Naphthenes	0.2197	0.0000	0.0086	0.0160	0.2142	0.2229
**Oxygenates**	**0.1076**	**0.0000**	**0.0012**	**0.0109**	**0.0305**	**0.1522**
Carboxylic acids	0.0263	0.0000	0.0002	0.0028	0.0052	0.0385
Alcohols	0.0351	0.0000	0.0003	0.0036	0.0086	0.0505
Ketones	0.0462	0.0000	0.0007	0.0045	0.0167	0.0632

Composition of OLP in Feed (%.area)					Recovery in Extract (Mass Fraction)	Recovery in Raffinate (Mass Fraction)
**Hydrocarbons**	**89.24**				**39.81**	**60.19**
Alkanes	**41.93**				34.21	65.79
Alkenes	**25.34**				52.63	47.37
Naphthenes	**21.97**				35.73	64.27
**Oxygenates**	**10.76**				**10.40**	**89.60**
Carboxylic acids	**2.63**				7.30	92.70
Alcohols	**3.51**				8.94	91.06
Ketones	**4.62**				13.28	86.72

Table S1: Process conditions, mass flow rates, gaseous fractions, recoveries of hydrocarbons and oxygenates in extract and raffinate, as well as chemical composition, expressed in solvent-free basis, of hydrocarbons and oxygenates of OLP in feed, top and bottom streams of absorber columns T-100 by simulation of OLP deoxygenation process in multistage countercurrent absorber columns using CO_2 as solvent, using Aspen-HYSYS 8.6 (Aspen One, 2015) at 333 K, 140 bar, and (S/F) = 17.

**ABSORBER T-102, (S/F) = 25**					**CO_2 (Free-Basis)**	**CO_2 (Free-Basis)**
Streams	**2**	**7**	**5**	**6**	**Extract**	**Raffinate**
Gas fraction	0.9252	1.0000	1.0000	0.0000		
**Temperature [C]**	77.8	77.8	93.5	100.8		
**Pressure [bar]**	140	140	140	140		
Molar Flow [kgmole/h]	18.89	36.12	49.90	5.11		
Mass Flow [kg/h]	880.94	1589.72	2227.47	243.19	**40.77**	**22.58**
**Composition of OLP (Mass Fraction)**						
Carbon-Dioxide	0.9281	1	0.9817	0.9072	0.0000	0.0000
n-Undecane	0.0026	0	0.0010	0.0000	0.0555	0.0001
n-Dodecane	0.0016	0	0.0006	0.0000	0.0335	0.0002
n-Tridecane	0.0034	0	0.0013	0.0004	0.0705	0.0039
n-Tetradecane	0.0075	0	0.0022	0.0071	0.1195	0.0765
n-Pentadecane	0.0114	0	0.0025	0.0186	0.1346	0.2001
n-Hexadecane	0.0024	0	0.0006	0.0028	0.0353	0.0299
n-Heptadecane	0.0013	0	0.0002	0.0025	0.0126	0.0269
n-Octadecane	0.0013	0	0.0001	0.0041	0.0038	0.0440
1-Decene	0.0005	0	0.0002	0.0000	0.0111	0.0000
cis-2-Decene	0.0001	0	0.0000	0.0000	0.0013	0.0000
1-Dodecene	0.0046	0	0.0018	0.0000	0.0991	0.0004
trans-2-Dodecene	0.0002	0	0.0001	0.0000	0.0041	0.0000
1-Tridecene	0.0001	0	0.0000	0.0000	0.0025	0.0001
1-Tetradecene	0.0004	0	0.0001	0.0002	0.0074	0.0017
(E)-9-Octadecene	0.0006	0	0.0001	0.0014	0.0047	0.0156
1-Pentadecene	0.0004	0	0.0001	0.0003	0.0056	0.0037
1-Heptadecene	0.0032	0	0.0006	0.0061	0.0322	0.0654
1-Nonadecene	0.0008	0	0.0001	0.0021	0.0055	0.0230
1,4-Undecadiene	0.0002	0	0.0001	0.0000	0.0036	0.0000
5-Icosene	0.0001	0	0.0000	0.0002	0.0006	0.0021
Isobutylcyclohexane	0.0001	0	0.0000	0.0000	0.0014	0.0000
n-Butylcyclohexane	0.0001	0	0.0000	0.0000	0.0015	0.0000
Cyclododecane	0.0003	0	0.0001	0.0001	0.0067	0.0009
n-Decylcyclopentane	0.0052	0	0.0013	0.0069	0.0715	0.0748
n-Nonylcyclopentane	0.0029	0	0.0009	0.0022	0.0497	0.0238
n-Nonylcyclohexane	0.0046	0	0.0011	0.0064	0.0612	0.0685
1-Ethyl-2 heptylcyclopropane	0.0002	0	0.0001	0.0000	0.0049	0.0000
1-Pentyl-2-propylcyclopropane	0.0006	0	0.0002	0.0000	0.0134	0.0000
1-Butyl-1-cyclohexene	0.0001	0	0.0001	0.0000	0.0028	0.0000
1-Hexylcyclopentene	0.0002	0	0.0001	0.0000	0.0049	0.0000
6-Dodecyne	0.0005	0	0.0002	0.0000	0.0109	0.0000
1-Heptyl-1-cyclohexene	0.0002	0	0.0001	0.0000	0.0043	0.0003
o-Cresol	0.0000	0	0.0000	0.0000	0.0008	0.0000
9-Octadecen-1-ol,-(Z)-	0.0036	0	0.0003	0.0102	0.0165	0.1102
2-Pentadecanone	0.0045	0	0.0007	0.0097	0.0402	0.1049
Palmitic acid	0.0018	0	0.0002	0.0044	0.0126	0.0476
Oleic acid	0.0010	0	0.0000	0.0031	0.0021	0.0338
1-Decylcyclohexene	0.0002	0	0.0001	0.0003	0.0033	0.0033
3-Hexadecene	0.0020	0	0.0005	0.0025	0.0290	0.0267
1-Nonil-CicloHexene	0.0012	0	0.0004	0.0011	0.0192	0.0114
**Composition of OLP in Feed (Mass Fraction)**					**Composition of OLP in Extract (Mass Fraction)**	**Composition of OLP in Raffinate (Mass Fraction)**
**Hydrocarbons**	**0.0610**	**0.0000**	**0.0170**	**0.0653**	**0.9278**	**0.7034**
Alkanes	0.0313	0.0000	0.0085	0.0354	0.4653	0.3815
Alkenes	0.0136	0.0000	0.0040	0.0129	0.2175	0.1388
Naphthenes	0.0160	0.0000	0.0045	0.0170	0.2449	0.1831
**Oxygenates**	**0.0109**	**0.0000**	**0.0013**	**0.0275**	**0.0722**	**0.2966**
Carboxylic acids	0.0028	0.0000	0.0003	0.0076	0.0147	0.0814
Alcohols	0.0036	0.0000	0.0003	0.0102	0.0174	0.1102
Ketones	0.0045	0.0000	0.0007	0.0097	0.0402	0.1049

**Composition of OLP in Feed (%.area)**					**Recovery in Extract (Mass Fraction)**	**Recovery in Raffinate (Mass Fraction)**
**Hydrocarbons**	**53.71**				**70.43**	**29.57**
Alkanes	**27.58**				68.77	31.23
Alkenes	**12.00**				73.89	26.11
Naphthenes	**14.12**				70.73	29.27
**Oxygenates**	**9.64**				**30.55**	**69.45**
Carboxylic acids	**2.44**				24.60	75.40
Alcohols	**3.19**				22.15	77.85
Ketones	**4.00**				40.87	59.13

Table S2: Process conditions, mass flow rates, gaseous fractions, recoveries of hydrocarbons and oxygenates in extract and raffinate, as well as chemical composition, expressed in solvent-free basis, of hydrocarbons and oxygenates of OLP in feed, top and bottom streams of absorber columns T-102 by simulation of OLP deoxygenation process in multistage countercurrent absorber columns using CO_2 as solvent, using Aspen-HYSYS 8.6 (Aspen One, 2015) at 350 K, 140 bar, and (S/F) = 25.

ABSORBER T-101, (S/F) = 38					CO_2 (Free-Basis)	CO_2 (Free-Basis)
Streams	**19**	**20**	**21**	**22**	**Extract**	**Raffinate**
Gas fraction	0.0000	1.0000	1.0000	0.0000		
**Temperature [C]**	60.0	60.0	101.1	67.2		
**Pressure [bar]**	140	140	140	140		
Molar Flow [kgmole/h]	0.10	12.81	7.01	5.90		
Mass Flow [kg/h]	22.71	563.86	311.94	274.62	**4.07**	**18.49**
**Composition of OLP (Mass Fraction)**						
Carbon-Dioxide	0.0065	1	0.9869	0.9327	0.0000	0.0000
n-Undecane	0.0001	0	0.0000	0.0000	0.0005	0.0001
n-Dodecane	0.0002	0	0.0000	0.0000	0.0007	0.0001
n-Tridecane	0.0038	0	0.0001	0.0002	0.0108	0.0023
n-Tetradecane	0.0758	0	0.0016	0.0044	0.1232	0.0660
n-Pentadecane	0.1987	0	0.0029	0.0132	0.2187	0.1959
n-Hexadecane	0.0296	0	0.0006	0.0018	0.0451	0.0265
n-Heptadecane	0.0267	0	0.0003	0.0018	0.0250	0.0273
n-Octadecane	0.0438	0	0.0001	0.0035	0.0082	0.0520
1-Decene	0.0000	0	0.0000	0.0000	0.0000	0.0000
cis-2-Decene	0.0000	0	0.0000	0.0000	0.0000	0.0000
1-Dodecene	0.0004	0	0.0000	0.0000	0.0015	0.0002
trans-2-Dodecene	0.0000	0	0.0000	0.0000	0.0001	0.0000
1-Tridecene	0.0001	0	0.0000	0.0000	0.0003	00001
1-Tetradecene	0.0017	0	0.0000	0.0001	0.0037	0.0012
(E)-9-Octadecene	0.0155	0	0.0001	0.0011	0.0105	0.0167
1-Pentadecene	0.0037	0	0.0001	0.0002	0.0062	0.0032
1-Heptadecene	0.0650	0	0.0008	0.0044	0.0629	0.0660
1-Nonadecene	0.0229	0	0.0002	0.0017	0.0129	0.0252
1,4-Undecadiene	0.0000	0	0.0000	0.0000	0.0000	0.0000
5-Icosene	0.0021	0	0.0000	0.0002	0.0013	0.0023
Isobutylcyclohexane	0.0000	0	0.0000	0.0000	0.0000	0.0000
n-Butylcyclohexane	0.0000	0	0.0000	0.0000	0.0000	0.0000
Cyclododecane	0.0008	0	0.0000	0.0000	0.0021	0.0006
n-Decylcyclopentane	0.0744	0	0.0014	0.0046	0.1039	0.0684
n-Nonylcyclopentane	0.0236	0	0.0006	0.0013	0.0439	0.0193
n-Nonylcyclohexane	0.0681	0	0.0012	0.0043	0.0922	0.0633
1-Ethyl-2 heptylcyclopropane	0.0000	0	0.0000	0.0000	0.0001	0.0000
1-Pentyl-2-propylcyclopropane	0.0000	0	0.0000	0.0000	0.0000	0.0000
1-Butyl-1-cyclohexene	0.0000	0	0.0000	0.0000	0.0000	0.0000
1-Hexylcyclopentene	0.0000	0	0.0000	0.0000	0.0000	0.0000
6-Dodecyne	0.0000	0	0.0000	0.0000	0.0002	0.0000
1-Heptyl-1-cyclohexene	0.0003	0	0.0000	0.0000	0.0008	0.0002
o-Cresol	0.0000	0	0.0000	0.0000	0.0000	0.0000
9-Octadecen-1-ol,-(Z)-	0.1096	0	0.0005	0.0085	0.0412	0.1255
2-Pentadecanone	0.1043	0	0.0011	0.0074	0.0849	0.1094
Palmitic acid	0.0473	0	0.0004	0.0035	0.0304	0.0514
Oleic acid	0.0336	0	0.0001	0.0027	0.0050	0.0402
1-Decylcyclohexene	0.0033	0	0.0001	0.0002	0.0047	0.0030
3-Hexadecene	0.0265	0	0.0005	0.0016	0.0392	0.0239
1-Nonil-CicloHexene	0.0113	0	0.0003	0.0006	0.0197	0.0096
**Composition of OLP in Feed (Mass Fraction)**					**Composition of OLP in Extract (Mass Fraction)**	**Composition of OLP in Raffinate (Mass Fraction)**
**Hydrocarbons**	**0.6987**	**0.0000**	**0.0109**	**0.0453**	**0.8385**	**0.6734**
Alkanes	0.3789	0.0000	0.0056	0.0249	0.4321	0.3701
Alkenes	0.1380	0.0000	0.0018	0.0093	0.1389	0.1389
Naphthenes	0.1818	0.0000	0.0035	0.0111	0.2674	0.1644
**Oxygenates**	**0.2948**	**0.0000**	**0.0021**	**0.0220**	**0.1615**	**0.3266**
Carboxylic acids	0.0810	0.0000	0.0005	0.0062	0.0354	0.0916
Alcohols	0.1096	0.0000	0.0005	0.0085	0.0412	0.1255
Ketones	0.1043	0.0000	0.0011	0.0074	0.0849	0.1094

Composition of OLP in Feed (%.area)					Recovery in Extract (Mass Fraction)	Recovery in Raffinate (Mass Fraction)
**Hydrocarbons**	**15.86**				**21.53**	**78.47**
Alkanes	**8.60**				20.46	79.54
Alkenes	**3.13**				18.06	81.94
Naphthenes	**4.13**				26.38	73.62
**Oxygenates**	**6.69**				**9.83**	**90.17**
Carboxylic acids	**1.84**				7.85	92.15
Alcohols	**2.49**				6.74	93.26
Ketones	**2.37**				14.61	85.39

Table S3: Process conditions, mass flow rates, gaseous fractions, recoveries of hydrocarbons and oxygenates in extract and raffinate, as well as chemical composition, expressed in solvent-free basis, of hydrocarbons and oxygenates of OLP in feed, top and bottom streams of absorber columns T-101 by simulation of OLP deoxygenation process in multistage countercurrent absorber columns using CO_2 as solvent, using Aspen-HYSYS 8.6 (Aspen One, 2015) at 333 K, 140 bar, and (S/F) = 38.
